# Supplementary figures and images for: Genomic rearrangements generate hypervariable mini-chromosomes in host-specific isolates of the blast fungus
Source: PLoS Genet. 2021 Feb 16;17(2):e1009386. doi: 10.1371/journal.pgen.1009386 (PMC7909708; doi:10.1371/journal.pgen.1009386)

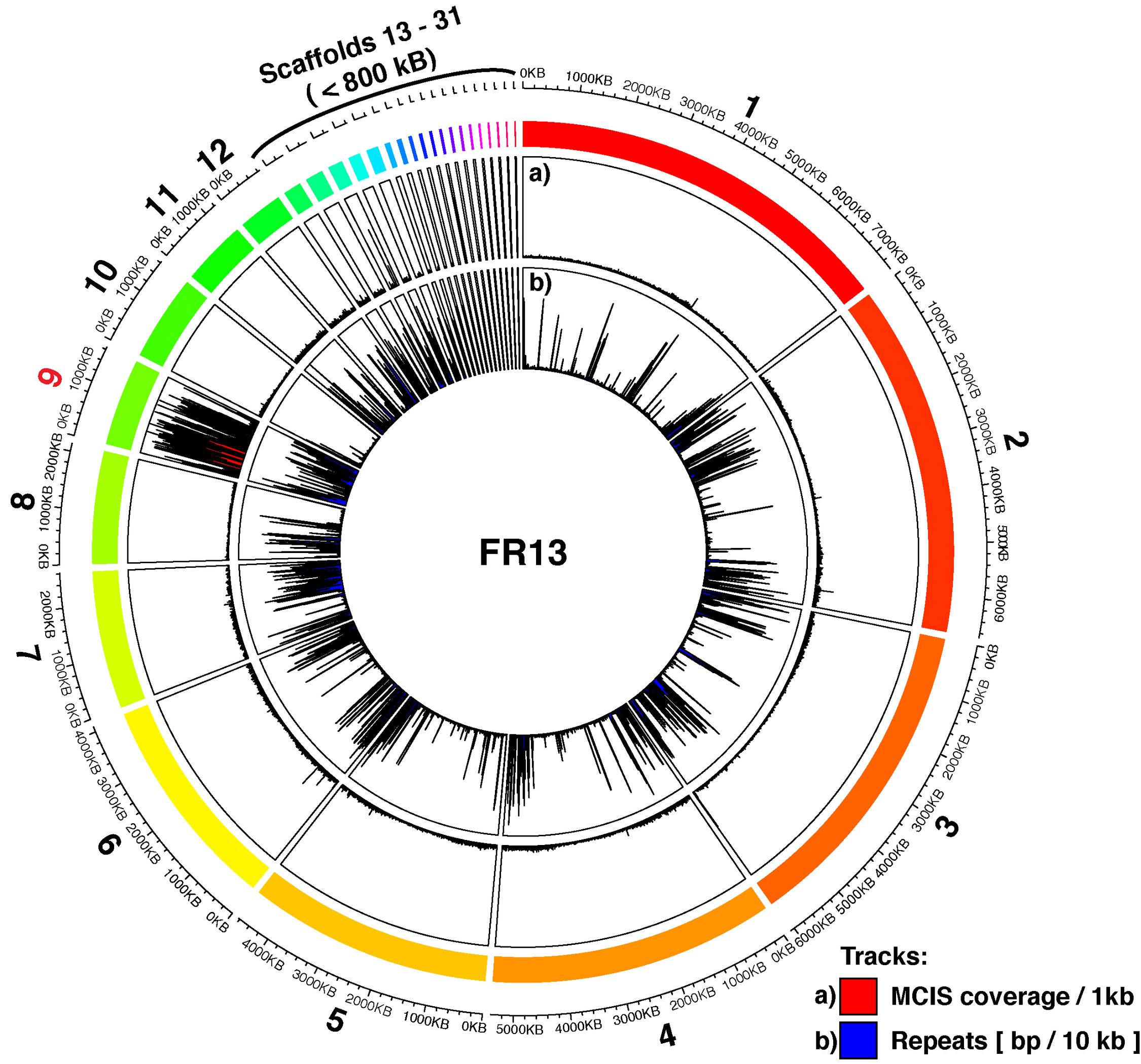

Supplement: S1 Fig — Circos plot of mini-chromosome isolation sequencing (MCIS) coverage and repeat content across the FR13 genome. Outer ring (rainbow colors): Scaffolds and scaffold sizes. Outer track (Red/Black): MCIS coverage per sliding window. Window size = 1000 bp; Slide distance: 500 bp. Y-axes: average coverage per 1 kb window; axis limits set to min/max coverage. Inner track (Blue/Black): Repeat content per sliding window. Window size = 10 kbp; Slide distance: 5 kbp. Y-axes: repeat content in bp per 10 kb window; axis limits set to zero to maximum. (TIF) [file pgen.1009386.s001.tif]

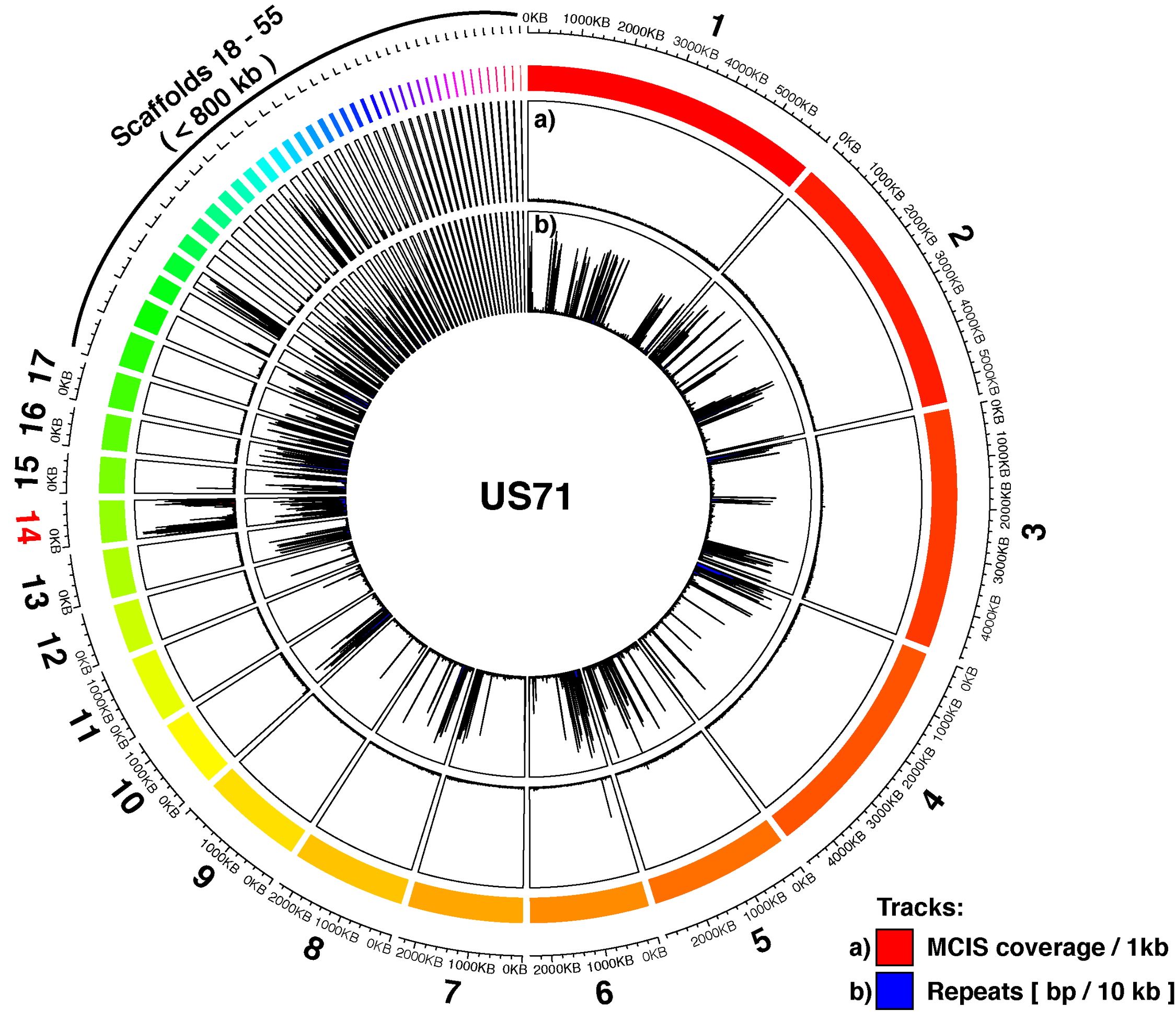

Supplement: S2 Fig — Circos plot of mini-chromosome isolation sequencing (MCIS) coverage and repeat content across the US71 genome. Outer ring (rainbow colors): Scaffolds and scaffold sizes. Outer track (Red/Black): MCIS coverage per sliding window. Window size = 1000 bp; Slide distance: 500 bp. Y-axes: average coverage per 1 kb window; axis limits set to min/max coverage. Inner track (Blue/Black): Repeat content per sliding window. Window size = 10 kbp; Slide distance: 5 kbp. Y-axes: repeat content in bp per 10 kb window; axis limits set to zero to maximum. (TIF) [file pgen.1009386.s002.tif]

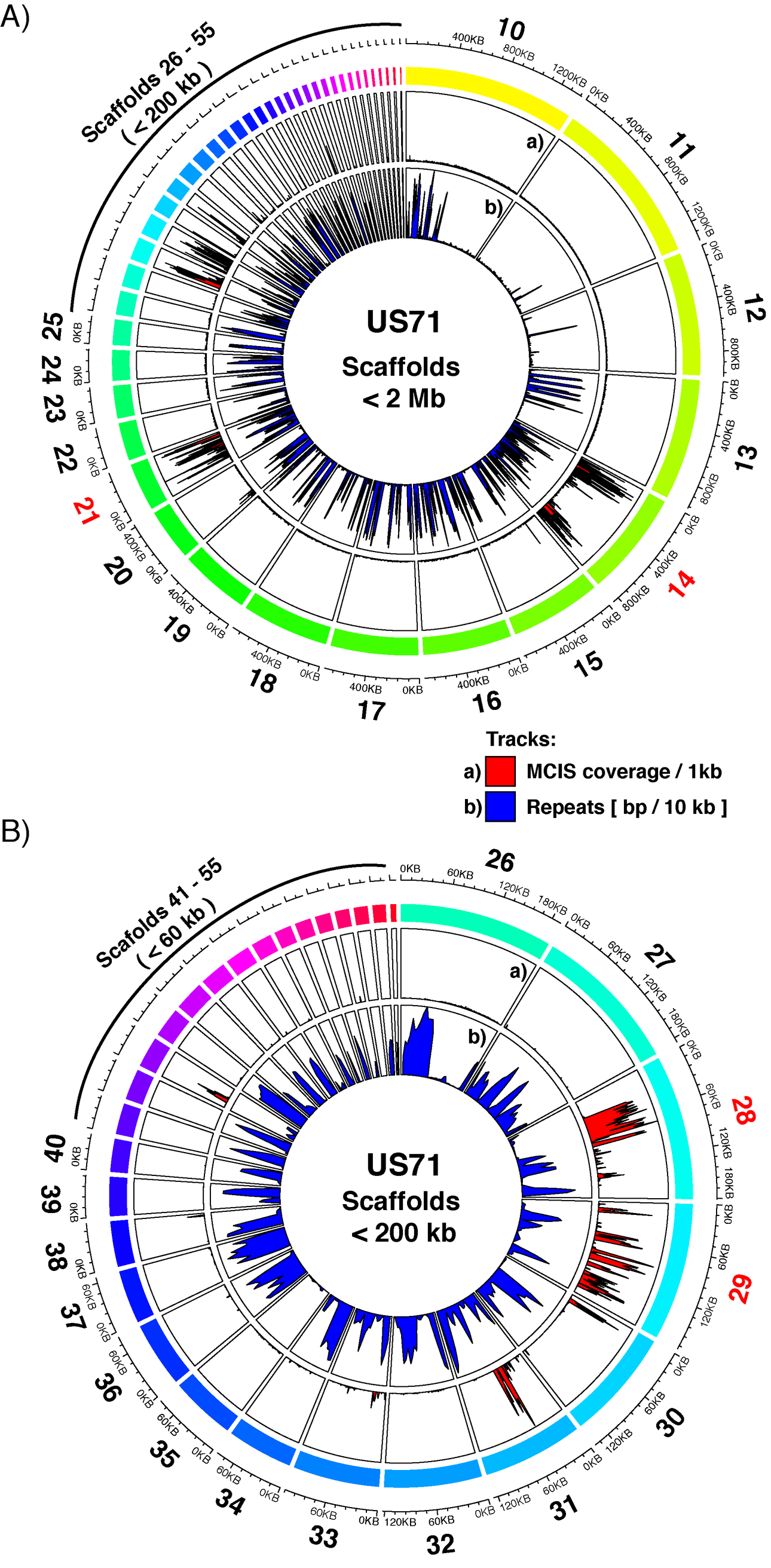

Supplement: S3 Fig — A) Circos plot of mini-chromosome isolation sequencing (MCIS) coverage and repeat content across US71 scaffolds < 2Mb. B) Circos plot of mini-chromosome isolation sequencing (MCIS) coverage and repeat content across US71 scaffolds < 200 kb. Outer ring (rainbow colors): Scaffolds and scaffold sizes. Outer track (Red/Black): MCIS coverage per sliding window. Window size = 1000 bp; Slide distance: 500 bp. Y-axes: average coverage per 1 kb window; axis limits set to min/max coverage. Inner track (Blue/Black): Repeat content per sliding window. Window size = 10 kbp; Slide distance: 5 kbp. Y-axes: repeat content in bp per 10 kb window; axis limits set to zero to maximum. (TIF) [file pgen.1009386.s003.tif]

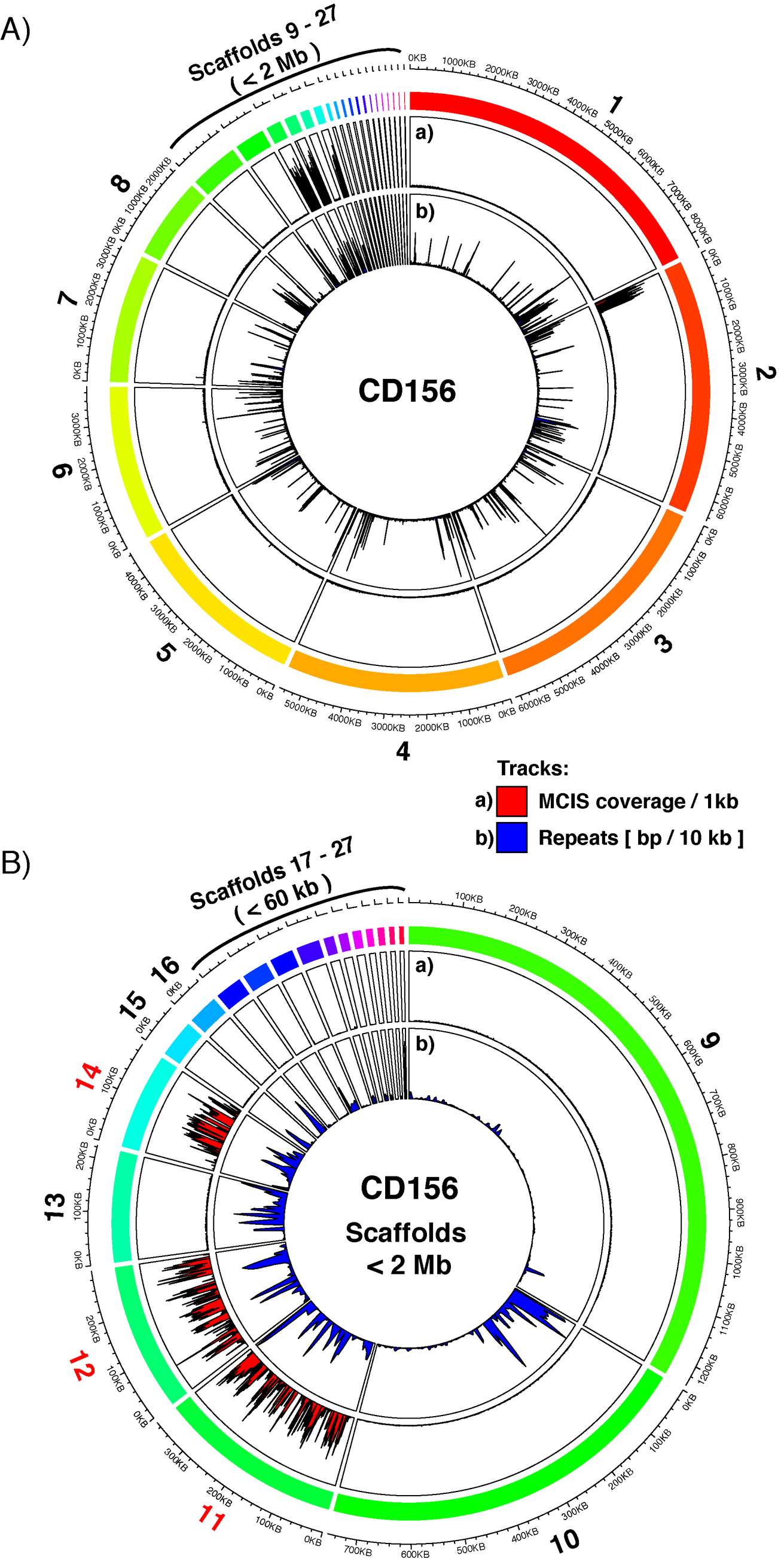

Supplement: S4 Fig — A) Circos plot of mini-chromosome isolation sequencing (MCIS) coverage and repeat content across the CD156 genome. B) Circos plot of mini-chromosome isolation sequencing (MCIS) coverage and repeat content across US71 scaffolds < 2Mb. Outer ring (rainbow colors): Scaffolds and scaffold sizes. Outer track (Red/Black): MCIS coverage per sliding window. Window size = 1000 bp; Slide distance: 500 bp. Y-axes: average coverage per 1 kb window; axis limits set to min/max coverage. Inner track (Blue/Black): Repeat content per sliding window. Window size = 10 kbp; Slide distance: 5 kbp. Y-axes: repeat content in bp per 10 kb window; axis limits set to zero to maximum. (TIF) [file pgen.1009386.s004.tif]

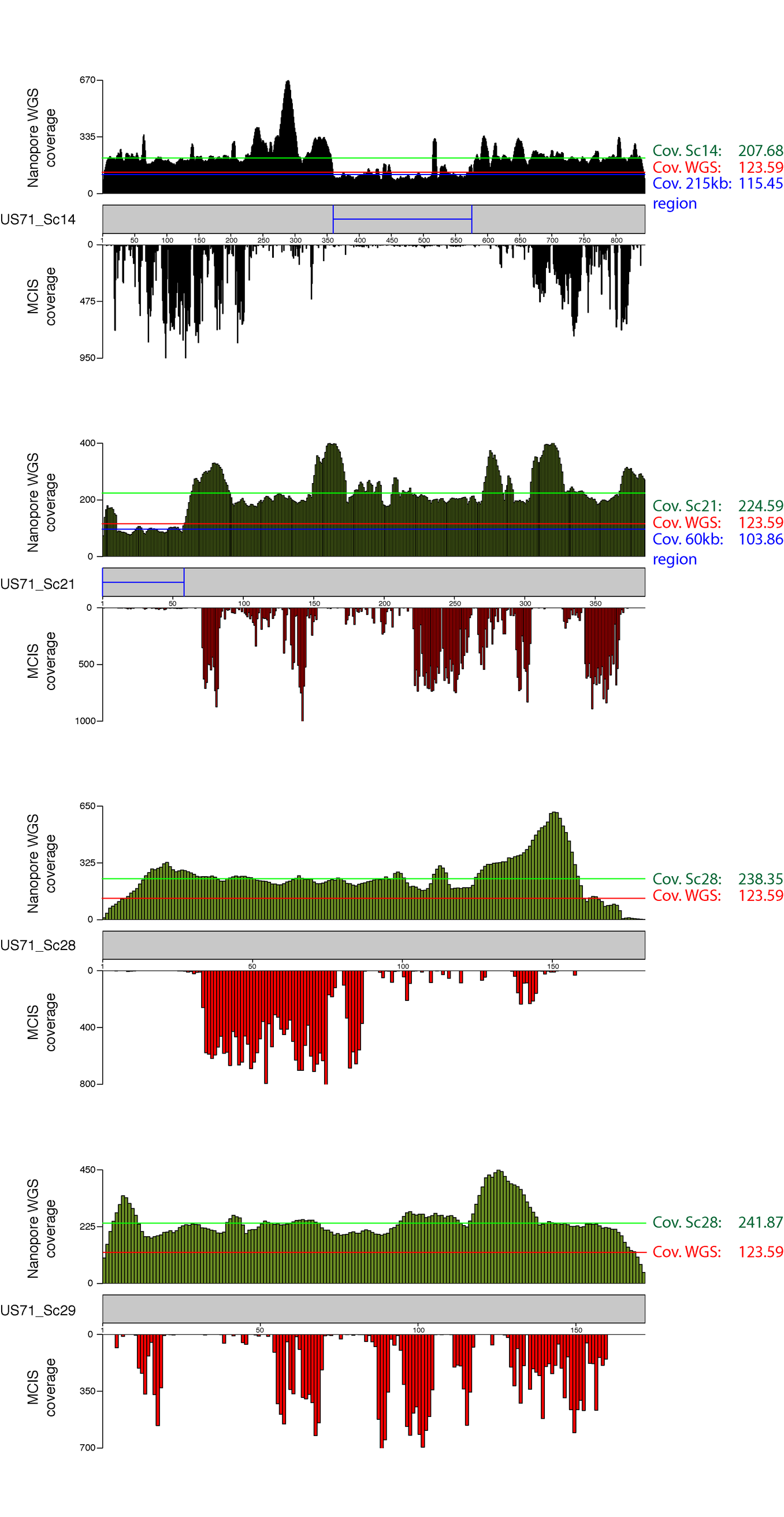

Supplement: S5 Fig — The upper panel of each plot shows the nanopore read coverage per 1 kb sliding window across proposed mini-chromosome contigs. Lower panels show mini-chromosome sequencing coverage per 1 kb sliding window. The red line indicates the average coverage across the whole genome. The green line shows the average coverage per scaffold and the blue line shows the average coverage in regions of reduced coverage in scaffolds 14 and 21. The regions are indicated by the blue bars. Axes limits were manually set to best represent the data range in each plot. (TIF) [file pgen.1009386.s005.tif]

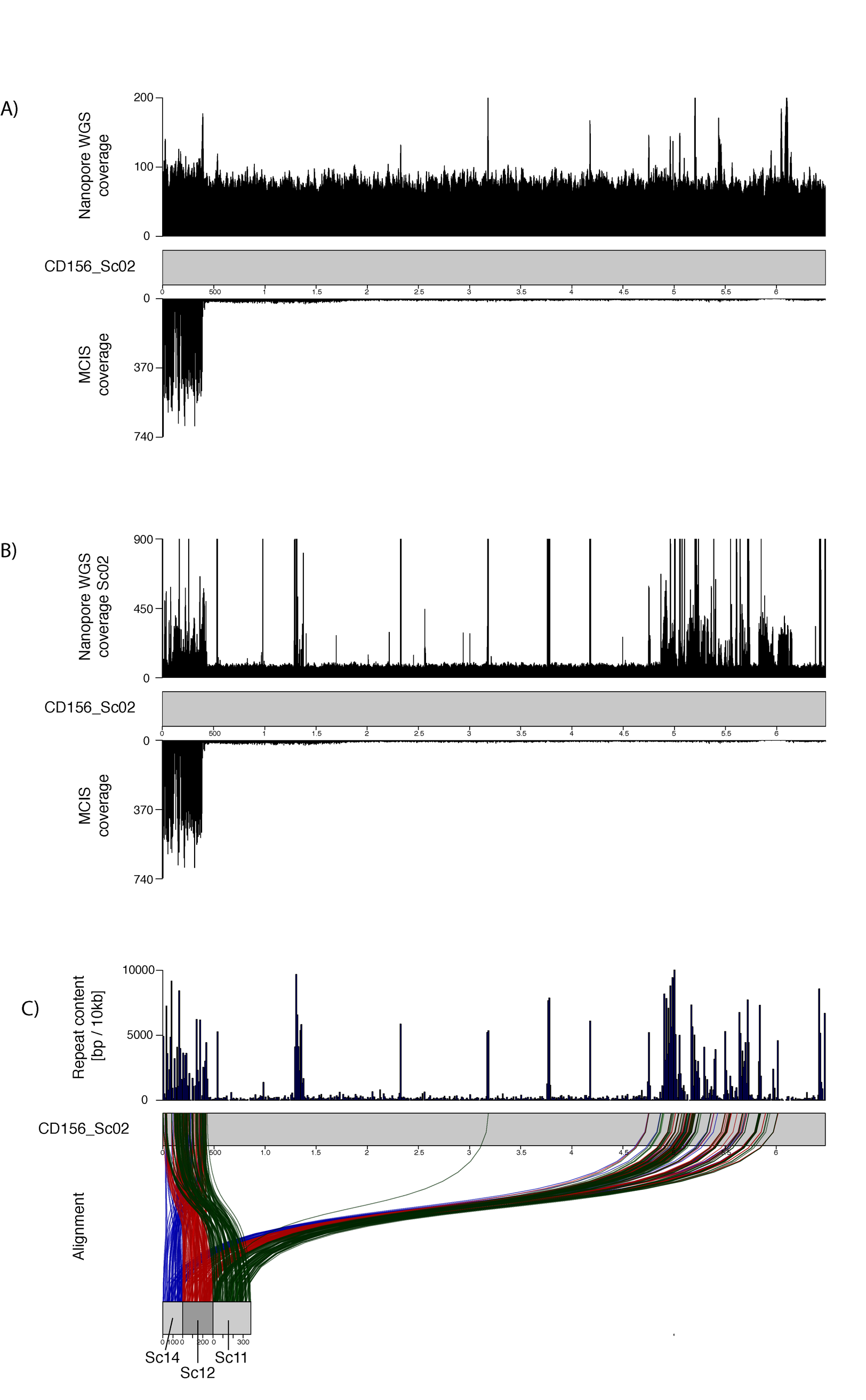

Supplement: S6 Fig — A) Nanopore read coverage across scaffold 2 after mapping to the whole genome sequence (upper panel) and mini-chromosome read coverage (lower panel) in CD156. B) Nanopore read coverage across scaffold 2 after mapping to the isolated scaffold 2 (upper panel) and mini-chromosome read coverage (lower panel) in CD156. C) Alignment of CD156 mini-chromosome scaffolds to scaffold 2 (upper panel) and repeat content of scaffold 2 (lower panel). Axes limits were manually set to best represent the data in each plot. (TIF) [file pgen.1009386.s006.tif]

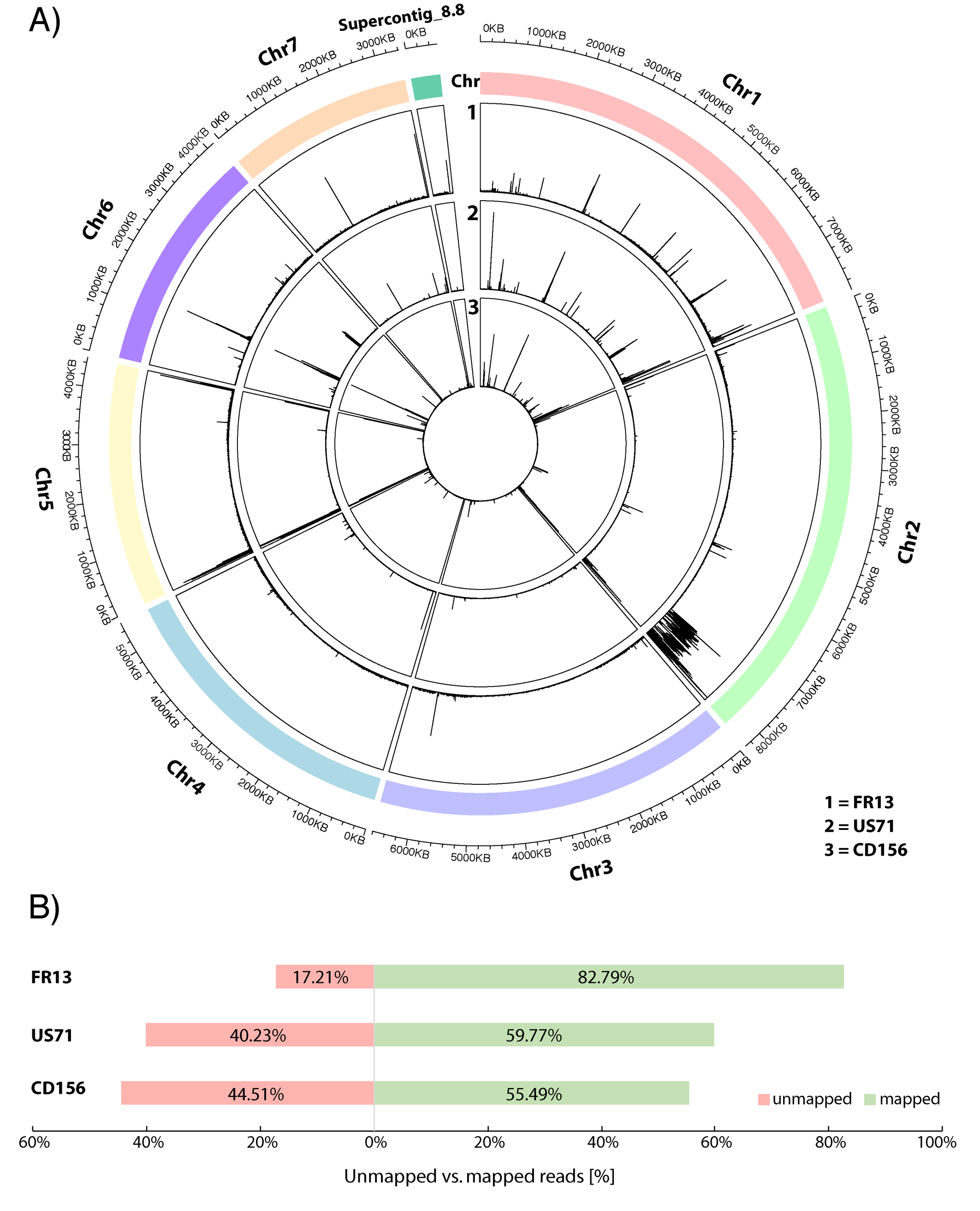

Supplement: S7 Fig — A) Circos plot of MCIS reads uniquely mapped against the 70–15 genome. Outer ring: 70–15 chromosomes and chromosome sizes. Tracks: 1. FR13 MCIS read depth, 2. US71 MCIS depth, 3. CD156 MCIS read depth. B) Relative amount of MCIS total reads that mapped to the genome of strain 70–15. Mapped reads shown in green, unmapped reads in red. (TIF) [file pgen.1009386.s007.tif]

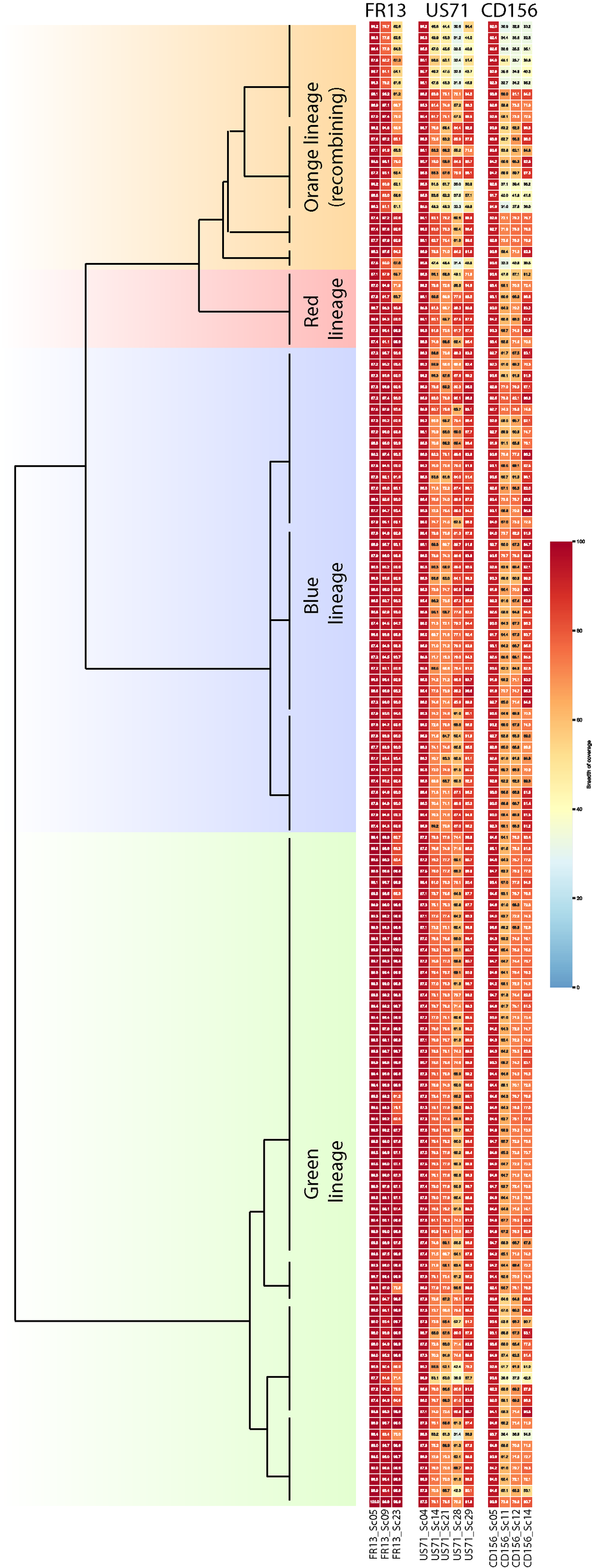

Supplement: S8 Fig — Breadth of coverage of each mini-chromosome scaffold and selected core-chromosome scaffolds after mapping of raw read data of 131 rice-infecting isolates. Isolate IDs are given on top of the columns. Scaffold IDs are given at the bottom. Left: Schematic representation of the rice-lineage phylogeny, adapted from Latorre et al., 2020. Sublineages are indicated by colors. The bottom row of the heatmap contains FR13 mapping data. (TIF) [file pgen.1009386.s008.tif]

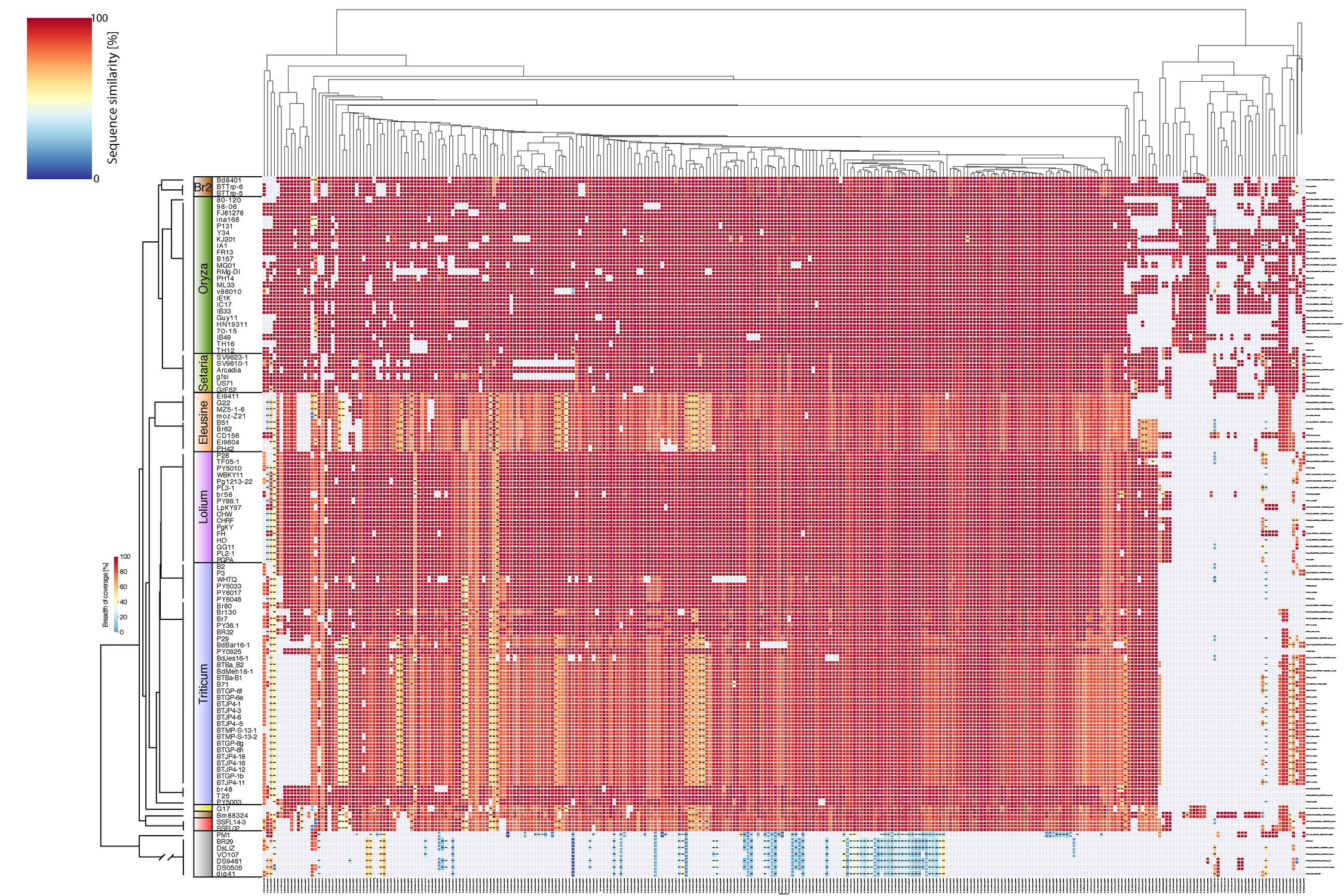

Supplement: S9 Fig — The heatmap show sequence similarity and presence/absence of genes encoded on the FR13 mini-chromosome across 10 genetic lineages of M. oryzae. Mini-chromosome encoded genes are hierarchical clustered on the x-axis. Isolate IDs are shown on the y-axis. Host-specific lineages are indicated on the left. (TIF) [file pgen.1009386.s009.tif]

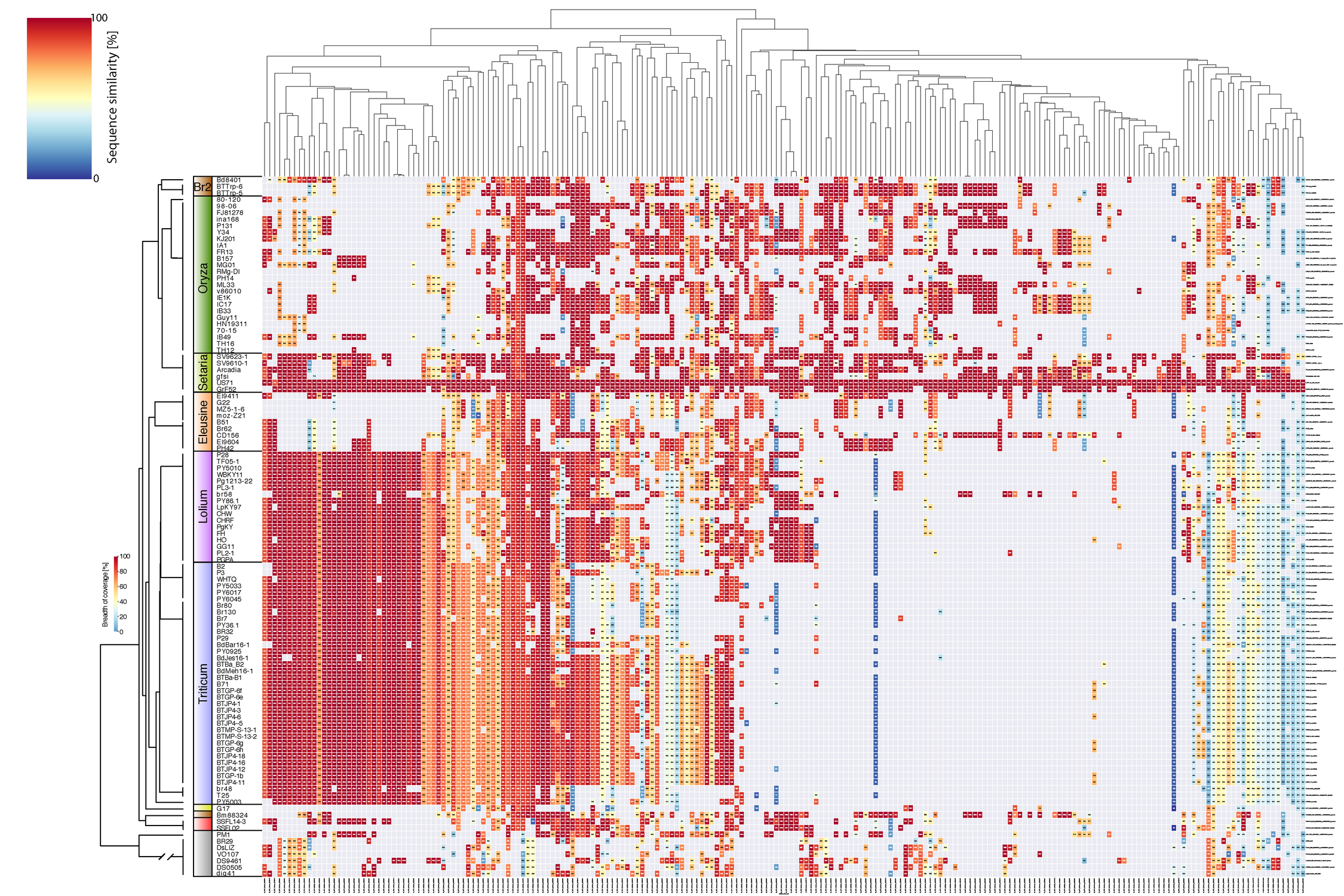

Supplement: S10 Fig — The heatmap show sequence similarity and presence/absence of genes encoded on the US71 mini-chromosome across 10 genetic lineages of M. oryzae. Mini-chromosome encoded genes are hierarchical clustered on the x-axis. Isolate IDs are shown on the y-axis. Host-specific lineages are indicated on the left. (TIF) [file pgen.1009386.s010.tif]

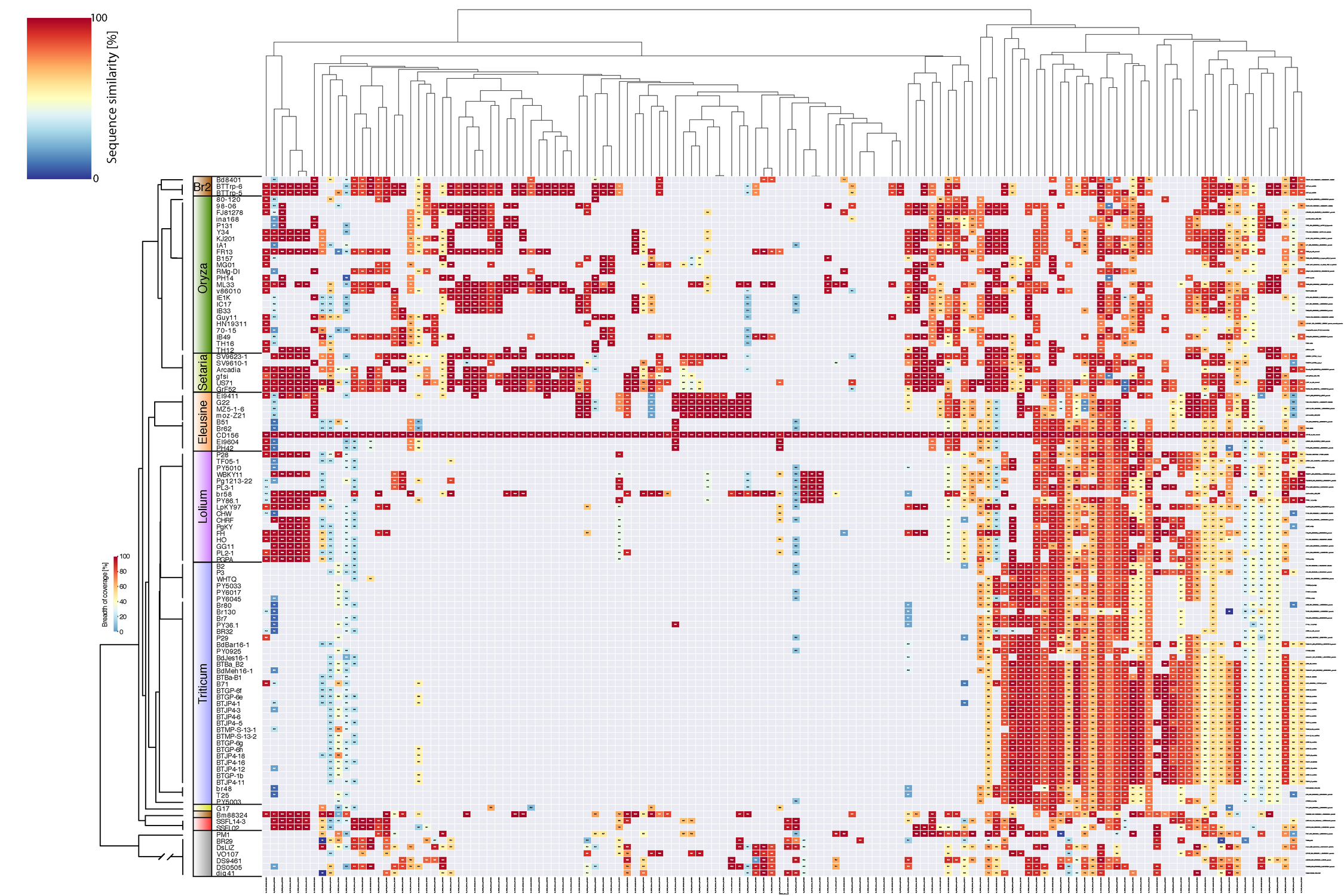

Supplement: S11 Fig — The heatmap show sequence similarity and presence/absence of genes encoded on the CD156 mini-chromosome across 10 genetic lineages of M. oryzae. Mini-chromosome encoded genes are hierarchical clustered on the x-axis. Isolate IDs are shown on the y-axis. Host-specific lineages are indicated on the left. (TIF) [file pgen.1009386.s011.tif]

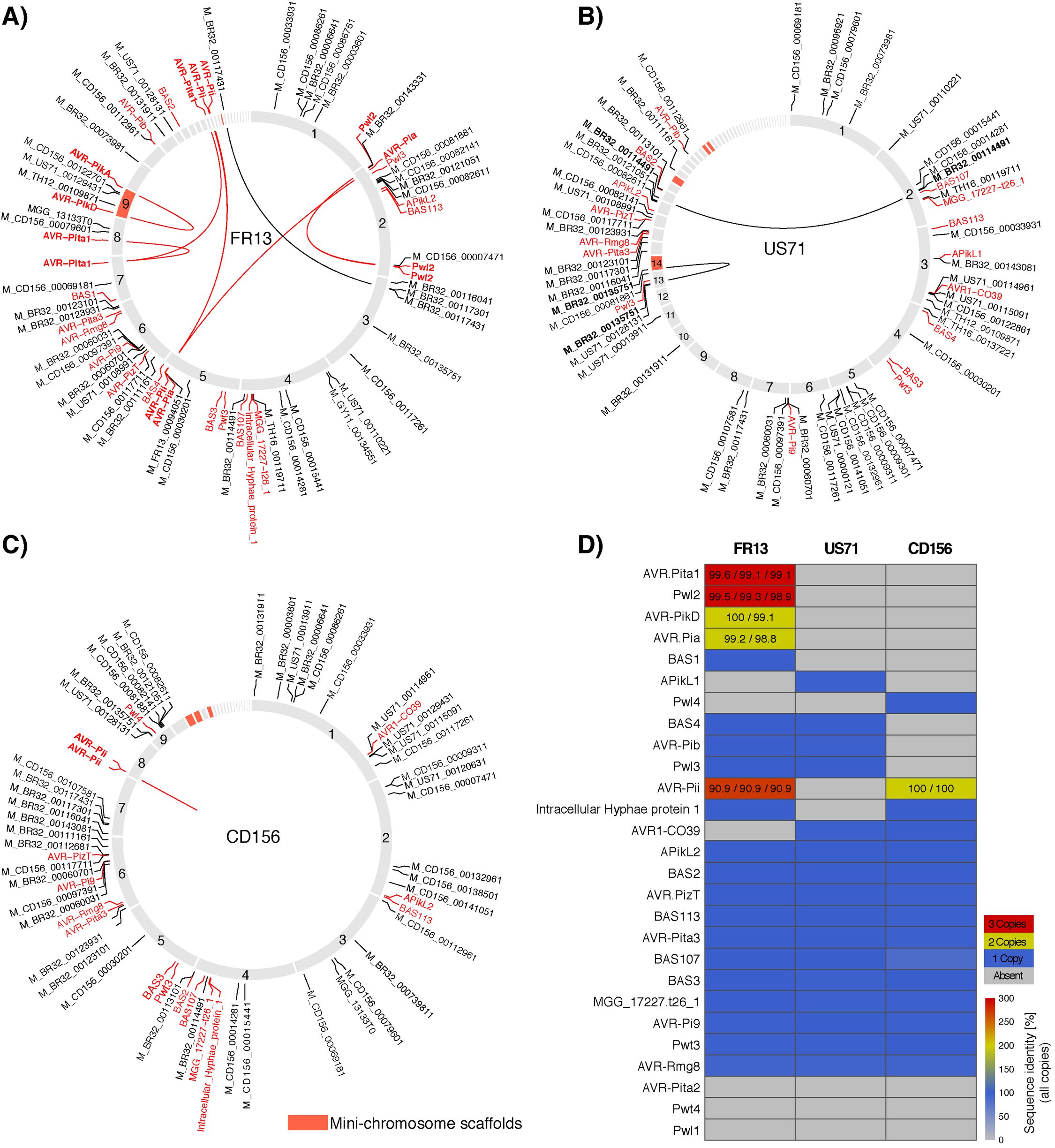

Supplement: S12 Fig — A) Position of effector genes in the FR13 genome. B) Position of effector genes in the US71 genome. C) Position of effector genes in the CD156 genome. Characterized effector genes are shown in red and predicted MAX-effectors are shown in black throughout A-C. Duplications are shown as lines in the center. Mini-chromosome scaffolds are shown in red, core-chromosomes in grey. D) Copy numbers of known effector genes in the genomes of isolates FR13, US71, and CD56. Absence shown in grey and presence shown in blue. Duplicated and triplicated genes are shown in yellow and red, respectively. Numbers in cells show the percentage identity of individual copies. (TIF) [file pgen.1009386.s012.tif]

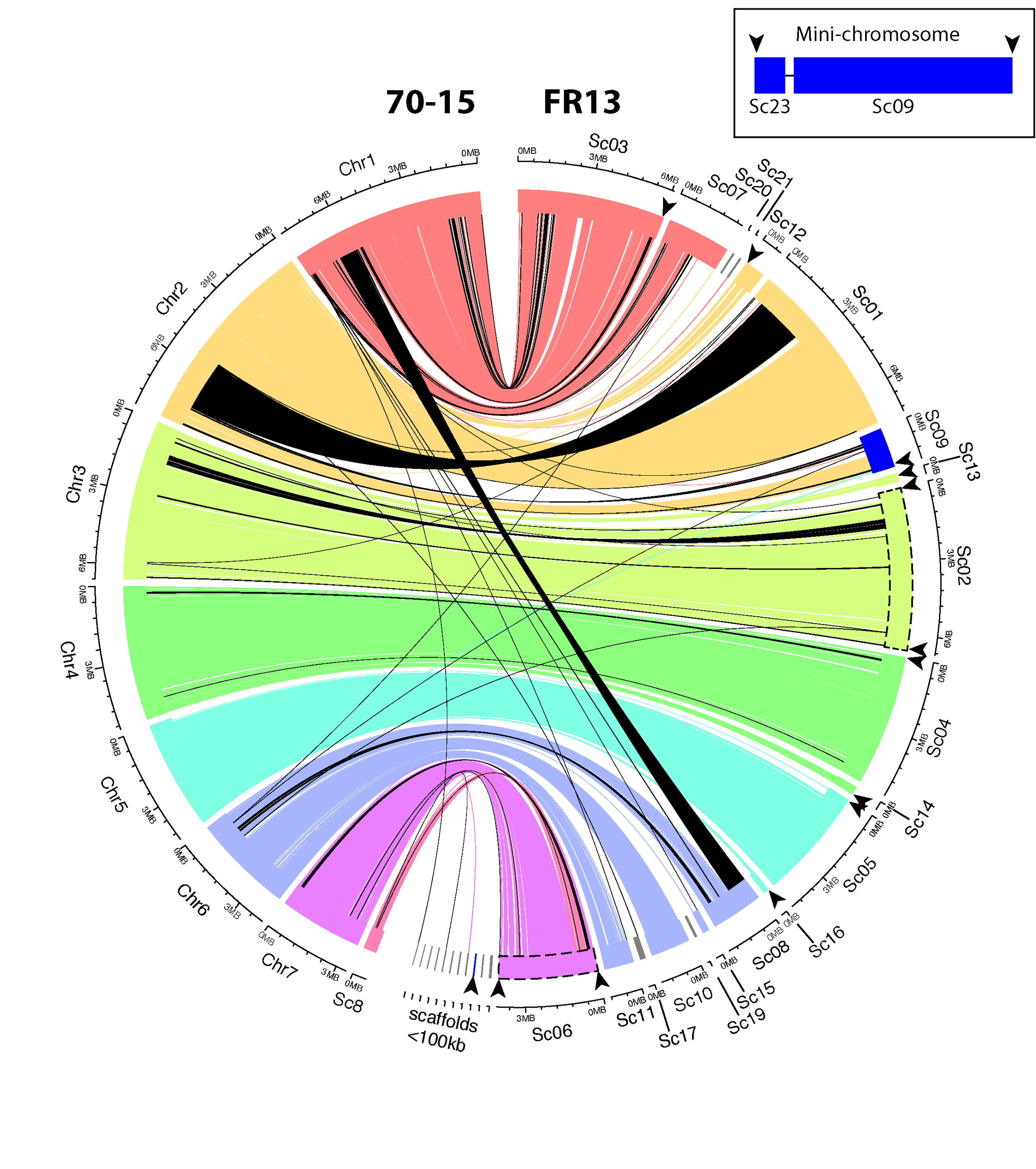

Supplement: S13 Fig — Alignments >10 kb between FR13 and the reference genome 70–15 are shown as genetic links after reordering the FR13 scaffolds according to the genome structure of 70–15 using MAUVE. Colors indicate the matching chromosomes and scaffolds between 70–15 and FR13. Colored genomic links represent alignments in forward direction. Black genomic links represent inverted alignments. Dashed lines show completely assembled chromosomes in FR13. Telomeric repeats are indicated by arrowheads. Mini-chromosome scaffolds are shown in blue. The proposed mini-chromosome structure is shown in the top right corner. (TIF) [file pgen.1009386.s013.tif]

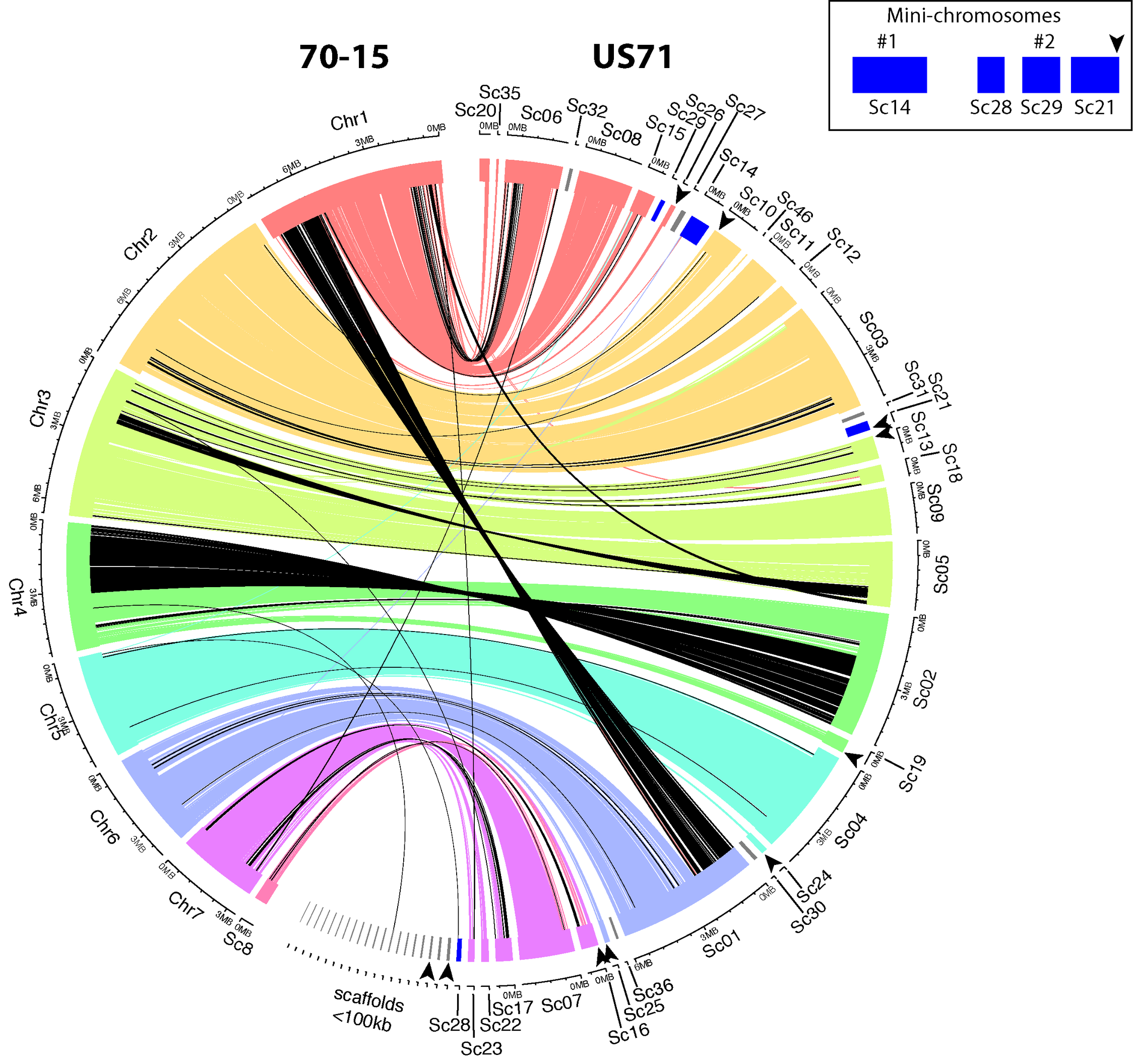

Supplement: S14 Fig — Alignments >10 kb between US71 and the reference genome 70–15 are shown as genetic links after reordering the US71 scaffolds according to the genome structure of 70–15 using MAUVE. Colors indicate the matching chromosomes and scaffolds between 70–15 and US71. Colored genomic links represent alignments in forward direction. Black genomic links represent inverted alignments. Telomeric repeats are indicated by arrowheads. Mini-chromosome scaffolds are shown in blue. The proposed mini-chromosome structure is shown in the top right corner. Note that, based on the current assembly, we cannot resolve the mini-chromosome of US71. (TIF) [file pgen.1009386.s014.tif]

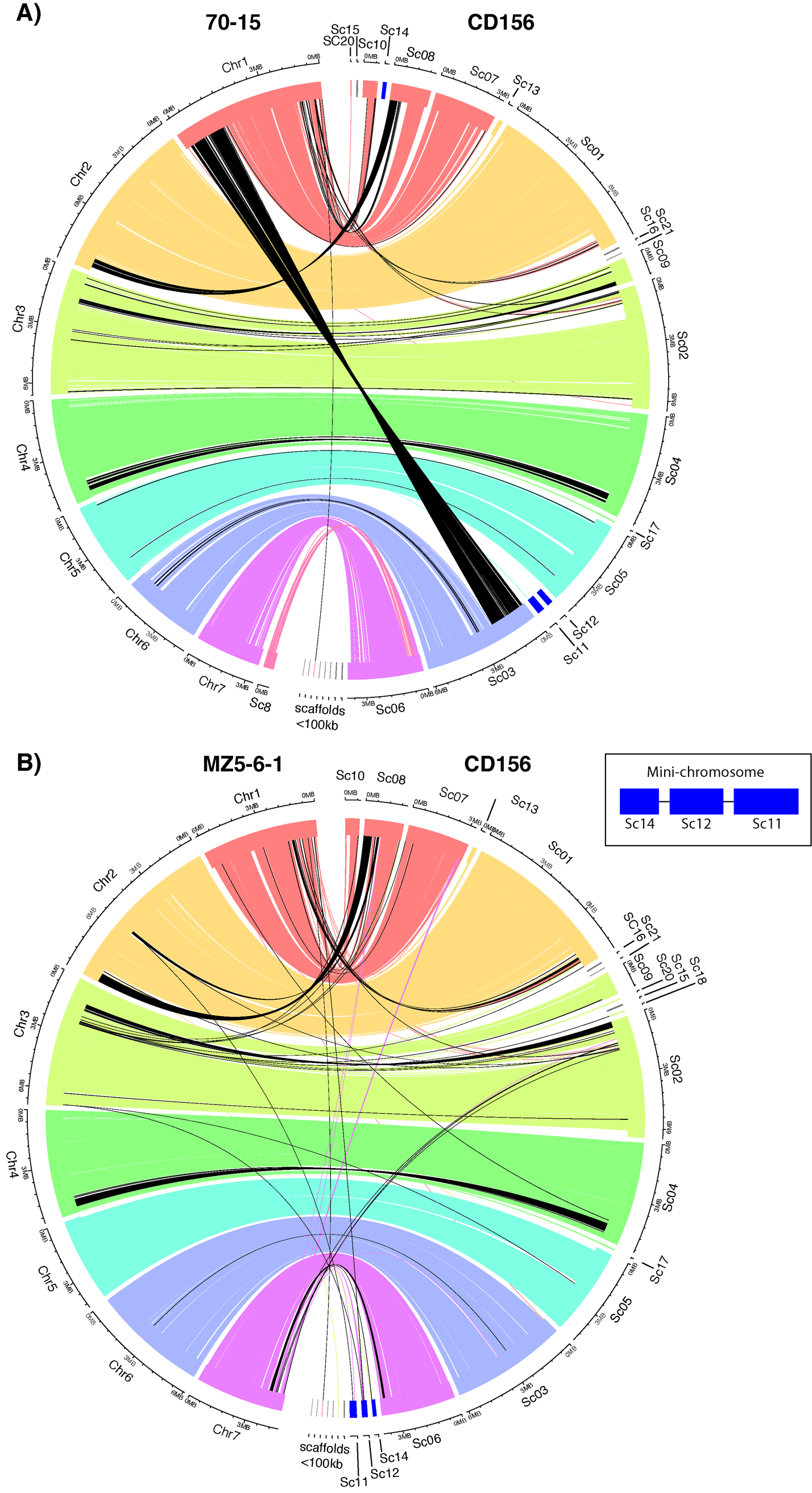

Supplement: S15 Fig — A) Alignments >10 kb between CD156 and the reference genome 70–15 are shown as genetic links after reordering the CD156 scaffolds according to the genome structure of 70–15 using MAUVE. Colors indicate the matching chromosomes and scaffolds between 70–15 and CD156. Colored genomic links represent alignments in forward direction. Black genomic links represent inverted alignments. Telomeric repeats are indicated by arrowheads. Mini-chromosome scaffolds are shown in blue. B) Alignments >10 kb between CD156 and the reference genome MZ5-6-1 are shown as genetic links after reordering the CD156 scaffolds according to the genome structure of MZ5-6-1 using MAUVE. Colors indicate the matching chromosomes and scaffolds between MZ5-6-1 and CD156. Colored genomic links represent alignments in forward direction. Black genomic links represent inverted alignments. Telomeric repeats are indicated by arrowheads. Mini-chromosome scaffolds are shown in blue. The proposed mini-chromosome structure based on the alignment to MZ5-6-1is shown in the top right corner. (TIF) [file pgen.1009386.s015.tif]

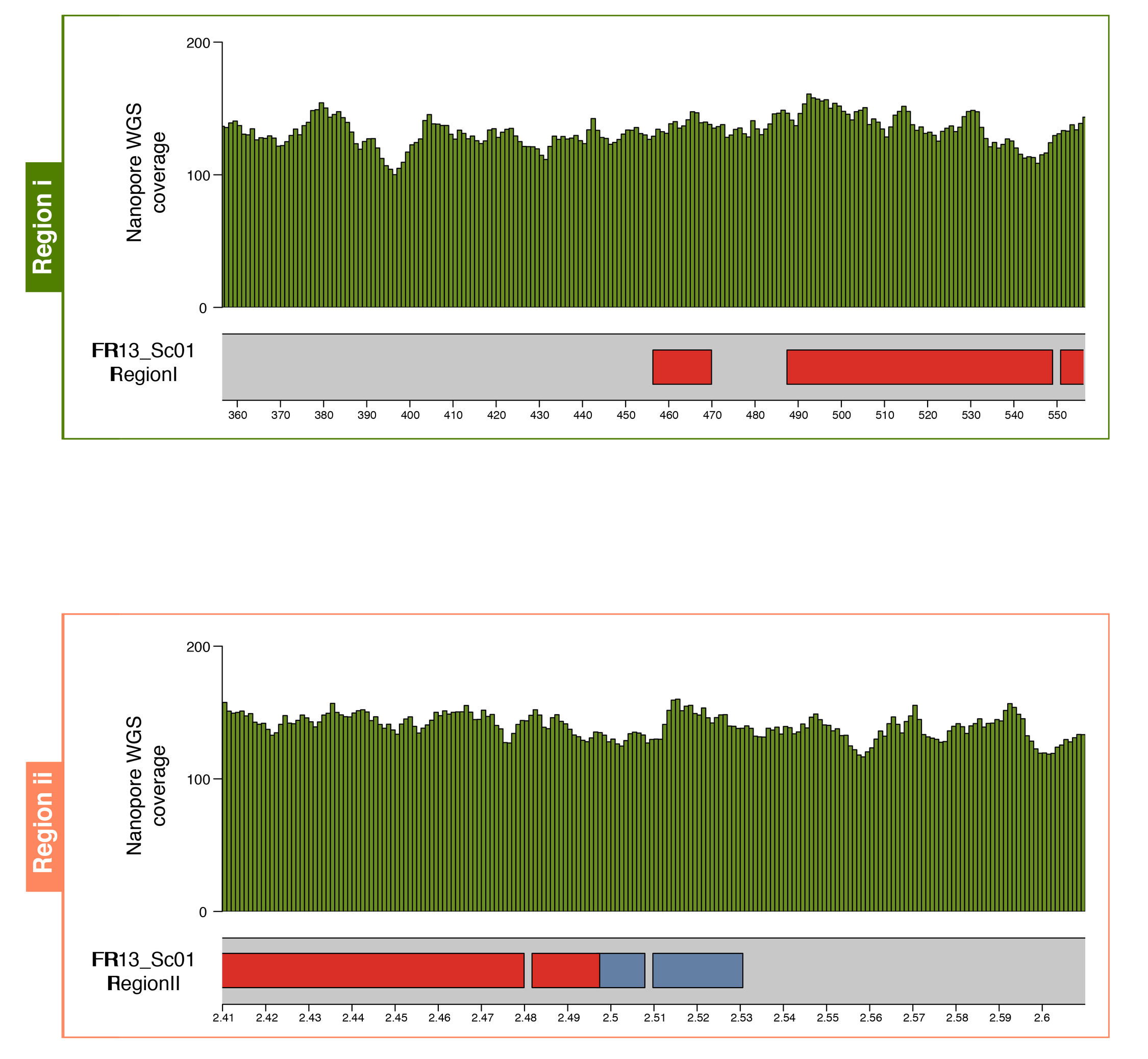

Supplement: S16 Fig — Nanopore coverage per 1 kb sliding window is shown in green bars. Alignments identified in Fig 6 are shown as red and blue rectangles. (TIF) [file pgen.1009386.s016.tif]

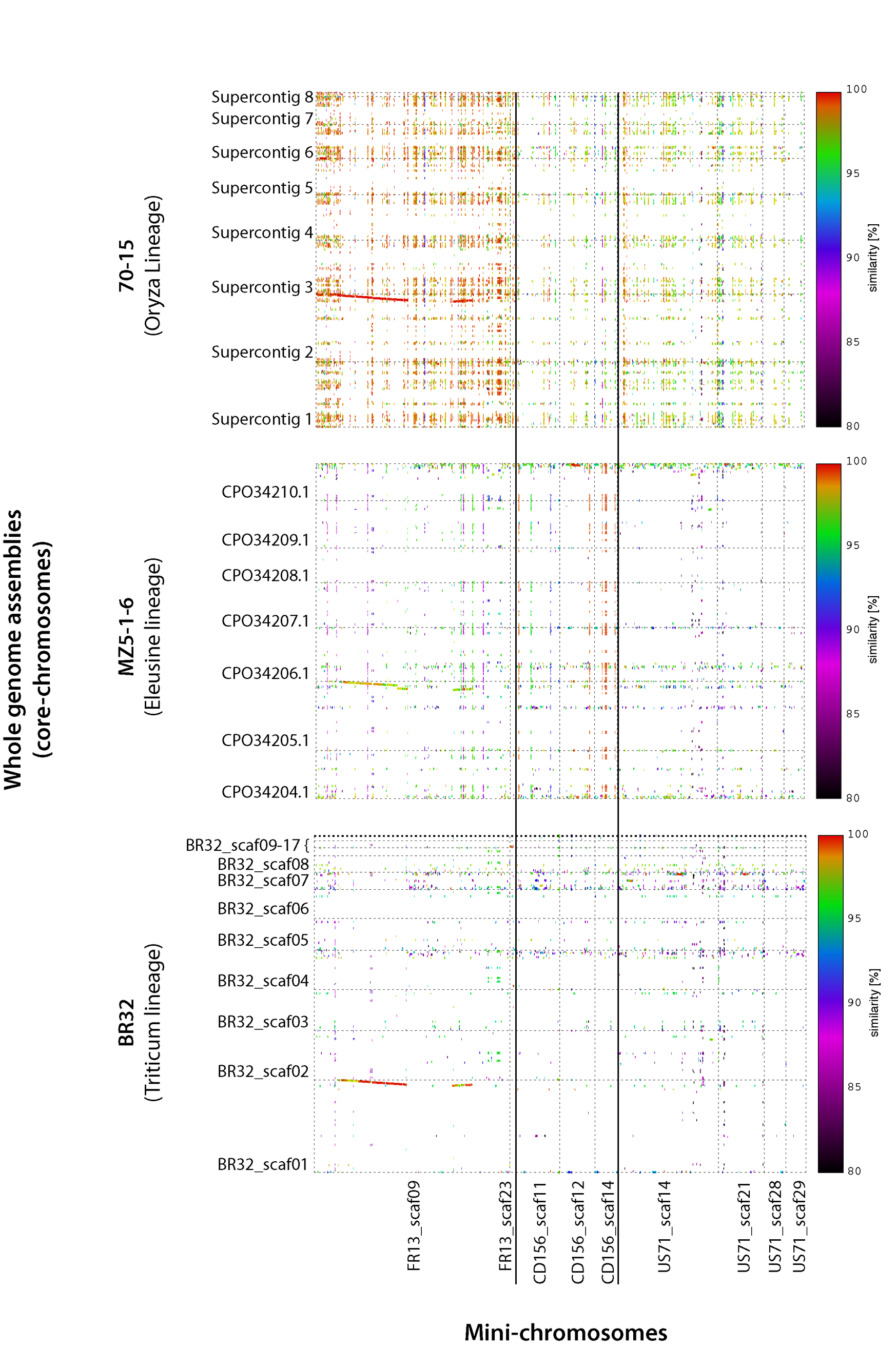

Supplement: S17 Fig — Raw alignment data of selected high quality whole genome assemblies against mini-chromosome scaffolds of FR13, US71 and CD156. X-axis: mini-chromosome scaffolds. Y-axis: whole genome assemblies of the isolates 70–15 (oryza lineage), MZ5-1-6 (eleusine lineage) and BR32 (triticum lineage). Dots show alignments. Lines indicate continuous alignments. Alignment color shows sequence similarity [%] between the query and the reference. Color scale = 80–100%. (TIF) [file pgen.1009386.s017.tif]
