# Supplementary material for: Genomic rearrangements generate hypervariable mini-chromosomes in host-specific isolates of the blast fungus
Source: PLoS Genet. 2021 Feb 16;17(2):e1009386. doi: 10.1371/journal.pgen.1009386 (PMC7909708; doi:10.1371/journal.pgen.1009386)
Supplement: Attachment — Submitted filename: Reviewer_comments_response_TL_sk_submitted.docx [file pgen.1009386.s025.docx]

**AUTHOR’S POINT-BY-POINT RESPONSE**

**Editor’s comments**

*“The reviewers liked the topic, but felt that the manuscript in its current form lacked the depth and novelty that we seek at PLoS Genetics.”*

We thank the reviewers and Dr. Xiaorong Lin for inviting us to submit a revision. We have taken the reviewer comments very seriously and have significantly revised the paper and included several new analyses.

We were initially very surprised by the editorial decision which seems to be driven mainly by the “lack of novelty” statement. This is contradictory to the PLOS editorial “scooping” policy outlined in this January 2018 article "The importance of being second" and described in the Instructions to Authors.

We, therefore, would like to make the reviewers aware that our paper is submitted under the PLOS “scooping” policy outlined in the article “The importance of being second” https://journals.plos.org/plosbiology/article?id=10.1371/journal.pbio.2005203

We totally concede that Peng et al. 2019 et al. preceded our paper and we cite their work 7 times. Also, we have compared our results to the results obtained in Peng et al. 2019 with the intention to generate more generalizable conclusions that are valid across lineage boundaries in this pathogen. We consider this a very important contribution as statements in Peng et al., 2019 were largely based on a single sample. Therefore, our study extends the work of Peng et al. which only focused on the wheat blast lineage whereas here we characterize mini-chromosomes from the hugely important rice blast pathogen and strains from two other host-specific lineages.

**Reviewer's Responses to Questions**

***Reviewer #1****: The manuscript “Genomic rearrangements generate hypervariable mini-chromosomes in host-specific lineages of the blast fungus” sequenced four field Magnaporthe oryzae isolates from four host species with the Nanopore long-read technology, producing four near finished genome assemblies. With the results from CHEF and MCIS, contigs derived from mini-chromosomes were identified. Characterization of sequences of mini-chromosomes found that they have lower levels of gene content and higher levels of repeats as compared to core-chromosomes, and mini-chromosomes seemed to have lineage-specific sequences. In addition, mini-chromosomes contain virulence-related loci, which are likely resulted from genomic rearrangements from core-chromosomes. The results from this study largely agree with the findings in a recent mini-chromosome paper, Peng et al., 2019 (reference 56). Most conclusions in the manuscript were well supported by data. The major concern for this manuscript is related to the speculation that mini-chromosomes emerged independently in different lineages, which was based on high levels of dissimilarity among mini-chromosomes from M. oryzae isolates from three host species. The high-level diversity of mini-chromosomes could be due to low levels of selective pressure, which allows high frequencies of base mutations and genomic rearrangements. It is not clear why independent emergence is necessary for the high-level diversity. Some detailed comments are listed below:*

We generally agree with the concerns of the reviewer, although, we want to point out that the currently available sequence data clearly shows sequence unrelated mini-chromosomes in these lineages. However, we agree that a small sample number as used in this manuscript cannot fully support this conclusion. We therefore changed the text to add that mini-chromosomes might emerge independently in lineages **or individual isolates**. We also included the statement “Future comparative analyses of natural populations of M. oryzae, beyond the few isolates we analyzed here, will shed light on the mechanisms of mini-chromosome evolution”

*L195-196: 17-55 are scaffold numbers rather than contig numbers*

Changed “contigs” to “scaffolds” throughout the manuscript.

*S1 fig: almost no blue color can be observed. Suggest adding notes on the figure to label each track or do other changes to make it clear.*

**Added track information** to the figures.

*About the hypothesis that US71 contains two near-identical mini-chromosomes, is it possible that some mini-contigs (identified as sequences from the mini-chromosome) are chimeric contigs from both core- and mini-chromosome? Enlargemet of S2 and S3 figures might provide the clue or reject this possibility.*

We addressed this issue by mapping nanopore whole genome sequencing data to the genomes and analysed all regions in question, including the rearrangements observed in FR13, the proposed segmental duplication from core-chromosome scaffold 2 in CD156 and the US71 mini-chromosome scaffolds.

For FR13 and CD156 these analyses substantiated our finding that core-genomic rearrangements and segmental duplications can contribute to mini-chromosome emergence.

For US71 this analysis is inconclusive and could be potentially a misassembly. We added sections to our results and discussion to flag and discuss the issues.

*P251-252: Again, this need to consider the possible assembly error.*

See above. We address this issue in the text and with new analyses.

*P274-275: “~900kb aligned on chr2 of 70-15” should be at the end of chr2 based on S5 fig. This is an interesting result, which should be mentioned here although it was described in the next paragraph.*

**Modified the sentence to:**

“However, only a 761 kb fragment of the FR13 mini-chromosome generated an alignment matching a ~900 kb region at the end of core chromosome 2 of isolate 70-15, whereas mini-chromosomes of US71 and CD156 did not generate significant alignments with the reference genome.”

*P287-293: The description is not clear. What does “largely overlapping regions” mean?*

**Modified paragraph to (lines 309-312):**

“We found that 82.78 %, 59.77 %, and 55.49 % of mini-chromosome reads of FR13, US71, and CD156, respectively, mapped to the same regions in the reference genome leading to local coverage peaks irrespective of the mini-chromosome reads used, indicative for unspecific mapping.”

**Added sentence** to highlight the higher degree of sequence conservation of mini-chromosomes of FR13 and 70-15 core-chromosomes:

“Conversely, only 17 % of mini-chromosome reads derived from the rice infecting isolate FR13 were strain specific, indicating higher levels of sequence conservation within the rice infecting isolates.”

*P304-307: The speculation is not well supported. Data indicated that mini-chromosomes are hypervariable. Not population data, particularly data from diverse isolated per lineages, here support that mini-chromosomes emerged independently in host-adapted lineages.*

**Modified sentence to (lines 328-330):**

“The difference in sequence similarity between core- and mini-chromosomes supports the hypothesis that mini-chromosomes represent isolate-specific genomic entities, possibly involved in host specialization and suggests that mini-chromosomes emerged independently in host-adapted lineages or individual isolates.”

We are confident that our data clearly shows that mini-chromosomes are specific to each isolate. We agree of course that the small sample size per lineage does not conclusively show whether or not mini-chromosomes are lineage- or isolate-specific. We do not make this general statement and have stated that population studies beyond the representative isolates we examined are needed. This said, to address the reviewer’s concern, we rephrased our hypothesis accordingly.

*P310-312: To map annotated genes to a new genome is not a satisfied approach for genome annotation. But I guess the authors just wanted to find homologs to an annotated gene database.*

Yes, the aim here was not to “re-annotate” the genomes, but to keep it comparable to previous GEMO annotation. In our opinion this was more useful as it enables comparison between the previous short read assemblies and the new nanopore assemblies. We used this approach to identify gene duplications that were unnoticed in previous assemblies. Additionally, we focused on previously characterized genes (e.g. AVR-Pik, Ace1) to support our conclusions as this seems to be most robust.

*P339-343: I don’t understand the reason for bootstrapping analysis. Data using all gene/repeats contents from all core and mini-chromosomal data are convincing. I don’t think bootstrapping is necessary.*

We wanted to ensure that our observation is not biased due to the small sample number (number of mini-chromosomes analysed =4) and size differences between core- (>40 MB) and mini-chromosomes (0.8-1.6 MB). The bootstrapping analysis shows that this is very unlikely a sampling issue and strengthens the analysis.

*P396-397: It is interesting that the number of secreted proteins varies dramatically among three strains. The numbers of genes on mini-chromosomes cannot completely explain this variation. I am just curious what implication this could provide.*

We agree that this is intriguing and we added text to our discussion (lines 656-662).

*L412-413: Assemblies of mini-chromosomes in this study cannot exactly know where are ends of mini-chromosomes, right? This statement probably needs to be revised.*

We thank the reviewer for the comment which prompted us to add this analysis to the manuscript. We mapped telomeric repeats to the assemblies and provide this information in several supplementary figures added to the manuscript. This analysis shows that mini-chromosomes contain telomeres and that the two copies of AVR-Pik are indeed encoded at both ends of the mini-chromosome of FR13.

*Which version of 70-15 reference genome was used? Is Supercontigs8.2 chr2?*

Added information in the main text (line194-196):

“…similar to the chromosome quality reference genome of strain 70-15 (assembly version 8, ensemble release 45; 98.2 %; Table 1).”

Added information in the material and methods section:

“Read mapping against the reference assembly version 8 of strain 70-15 and visualization was carried out as described above.”

And:

“The supercontig number in assembly version 8 of isolate 70-15 corresponds to the chromosome number and supercontig_8.8 contains unmapped sequence.”

***Reviewer #2:*** *The manuscript “Genomic rearrangements generate hypervariable mini-chromosomes in host-specific lineages of the blast fungus” by Thorsten Langner and colleagues described mini-chromosomes in four lineages of the blast fungus. In agreement with many reports from other pathosystems, such as Fusarium, that these mini-chromosomes differ from core chromosomes with all distinct characteristics: high repeat, low gene content, and lack house-keeping genes.*

We only report mini-chromosomes in 3 lineages and not four as stated by the reviewer. Further, we did not claim that mini-chromosomes in the blast fungus lack house-keeping genes. Our analysis (e.g. Fig 5) clearly shows that mini-chromosomes share sequences with core-chromosomes of other isolates which prompted our conclusion that mini-chromosomes emerge from core-genomic rearrangements.

*The discovery is interesting, but not quite novel. Even though different terms were used, mini-chromosomes had been a focus for many other pathosystems, such as Zymoseptoria and Fusarium just to name a couple. Also, a paper published in PLOS Genet 2019 “Effector Gene Reshuffling Involves Dispensable Mini-chromosomes in the Wheat Blast Fungus” by Dr. Valent and colleagues already extensively documented such a phenomenon in a blast fungal genome. Unfortunately, this work was not even cited in this manuscript and the mini-chr reported in that paper was not included in the comparison.*

We were very surprised by this statement, which indicates the reviewer didn’t carefully read the paper.

We cite the study in question (Peng et al., 2019 “Effector Gene Reshuffling Involves Dispensable Mini-chromosomes in the Wheat Blast Fungus”) 7 times (!) in our manuscript and compare and discuss our results extensively in the context of this study.

We also refer 3 times to Peng et al., 2019 in our cover letter in which we clearly state that our research is complementary to and extends the findings of Peng et al. 2019 and should be reviewed under these criteria.

In addition, as clearly stated in the cover letter, we submitted the paper according to the PLOS “scooping” policy outline in this January 2018 article "The importance of being second" and described in the Instructions to Authors. The reviewer needs to be informed and instructed that novelty is not of relevance here. In fact, because PLOS Genetics published Peng et al. 2019 then the journal must have deemed the topic of *M. oryzae* mini-chromosomes novel and relevant enough.

Our paper validates, complements, and extends the findings reported in PLOS Genetics by Peng et al. in September 2019 in several ways:

1. We extend the mini-chromosome identification from only one lineage of *M. oryzae* to three host-specific isolates, thus expanding the concept beyond wheat blast strains and setting the stage for comparative genomics of mini-chromosomes in populations of the blast fungus.
2. We report the first sequence of a mini-chromosome of a rice blast isolate. This pathogen is viewed as the most destructive fungal plant pathogen.
3. We validate common, genetic features in several independent mini-chromosomes. This strengthens the conclusions reached Peng et al. and shows that all mini-chromosomes sequenced to date have a similar sequence composition.
4. We provide additional evidence that structural rearrangements facilitate mini-chromosome emergence and might facilitate adaptive evolution of virulence-related genes in plant pathogens.
5. We show that mini-chromosomes of host-specific strains are highly variable and probably emerge independently as these lineages have diversified throughout evolution.

*The author concluded that mini-chromosomes were emerged from structural rearrangements of core-chromosomes. I feel this conclusion is not solidly supported. Here are two reasons:*

*1) Comparison between mini-chr with core-chr revealed some similarity, but more than 50% are not accountable.*

*a total of 42,974 genes between all three isolates, which can be formed into 12,900 orthogroups.*

*9,003 were conserved across all three*

*3,897 were absent in at least one strain*

*1,827 that were conserved between two*

*2,070 were unique to single strains.*

*50% have no functional annotation*

We do not follow the logic of reviewer 2: We concluded that mini-chromosomes emerge from structural genomic rearrangement based on synteny, sequence conservation, and the observation that some mini-chromosome genes that are found on core-chromosomes in other isolates (supporting the hypothesis raised in Peng et al., 2019 which was based on only 1 isolate). The analysis of orthogroups has nothing to do with this statement.

Also, the reviewer is mixing up statements: “50% have no functional annotation” this data comes from the domain analysis of mini-chromosome encoded proteins and has nothing to do with the overall sequence similarity or orthogroups.

*2) current data can’t differentiate two hypotheses of a) two mini-chromosome of the same size b) segmental duplication and translocation based on the observation of increased coverage of CD156. If second hypothesis is correct (based on our experience, it is very possible), then the match could be the reintroduction of mini-chr content to the core, in the contrast of the conclusion presented by the authors.*

*This definitely needs to investigate further.*

Again, the reviewer is mixing up unrelated information. We raise the possibility that **US71** **contains 2 mini-chromosomes** of same size (based on the size of mini-chromosome contigs = double the size that we observed in chef gel). We later hypothesize that **segmental duplication** contributed to mini-chromosome emergence in **CD156**. Also, we don’t report any “increased coverage” for CD156, only the observation that mini-chromosome reads also map to contig2 in this isolate (S4 Fig).

That said, in our revised manuscript we added several new analyses that support our hypotheses about segmental duplication of core-chromosome scaffold 2 and the mini-chromosome of isolate CD156. We also analyzed the mini-chromosome scaffolds of isolate US71. This analysis, although not fully conclusive, points towards a possible duplication of segments of the US71 mini-chromosome scaffolds 14 and 21. We added several additional figures and discussed this issue.

***Reviewer #3****: The authors assess the mini-chromosomes in several lineages of M. oryzae. Large fragment genomic sequences were obtained by nanopore sequencing, and mini-chromosome specific reads obtained from CHEF gel separated chromosomes. Three of the four host specific lineages of M. oryzae had mini chromosomes, which were found to have several characteristics significantly different than the “core” chromosomes including a reduced gene density and an increase in repetitive sequences. These mini-chromosomes appear to be enriched in genes encoding putative virulence factors. Synteny analysis indicated regions of synteny around the ACE1 locus, a known virulence factor; however the lack of conservation of these mini-chromosomes led to speculation that they arose independently.*

*While the improved assembly and reanalysis of the four M. oryzae genomes is a nice addition, overall the manuscript adds little new information to what was previously known about supernumerary chromosomes and lacks experimental support. Are any of these mini-chromosomes able to be lost? Do they influence the host-specificity of each of the lineages?*

The scope of the manuscript was to identify and analyse mini-chromosomes in independent lineages and thus extending and complementing the knowledge gained in wheat blast isolates from the study of Peng et al. 2019. Here again, we wonder whether the reviewer was made aware that our manuscript should be considered in light of the PLOS “scooping” policy outline in this January 2018 article "The importance of being second". Clearly, without this knowledge, the reviewer is indeed correct in viewing our paper as lacking in novelty relative to Peng et al. 2019.

We refer the reviewer to the text above that describes how our paper complements Peng et al. and is in fact an ideal example of complementary research paper that adds value to the earlier paper.

Therefore, we think the additional experiments asked for here are beyond the scope of the manuscript, especially when the content of our paper and depth of analyses are compared to Peng et al. 2019 published just months ago in PLOS Genetics and evaluated by the same Editor.

We also want to repeat that we specifically requested in our cover letter that this should be evaluated as a complementary research article in accordance with PLOS editorial policies. It’s a pity that the reviewers were not instructed and alerted in this regard.

*1. Do other field isolates in the same host-specific lineages have the same (or similar) mini-chromosomes? Sequencing the CHEF-extracted mini-chromosomes would add support for many of these conclusions.*

We agree with reviewer 3 that this is a very important question and we even discuss this question in our manuscript. However, identification of mini-chromosomes requires long read whole genome assemblies and separate mini-chromosome sequencing for each isolate. Therefore, we think that a population genomics analyses of mini-chromosomes is beyond the scope of this study.

In our manuscript we provide evidence that a substantial amount of the FR13 mini-chromosome encoded sequence (>900 kb) is shared between the closely related isolates FR13 and 70-15. Both of these isolates belong to the rice-infecting lineage of M. oryzae. This degree of conservation is not present between the mini-chromosomes of the more divergent strains US71 (Setaria-infecting lineage) and CD156 (Eleusine-infecting lineage). This data is shown in Fig S5 in our manuscript. **In response to the reviewer comment we added whole genome comparisons to our manuscript** and found that similarly, CD156 mini-chromosome scaffolds produce several alignments of >10 kb when compared to the eleusine lineage reference assembly of isolate MZ5-6-1 that are missing when compared to the rice blast reference assembly of isolate 70-15.

We thank the reviewer for these remarks and added several whole genome comparisons to our manuscript in response to the reviewer comments. These include whole genome alignments, contig-reordering, and annotation of canonical, telomeric repeats relative to the genome structure of the reference assemblies of isolates 70-15 and MZ5-6-1 (used as a reference for the isolate CD156 which belongs to the eleusine infecting lineage).

Taken together, this data supports our conclusion that mini-chromosomes contain isolate or lineage specific sequences with higher degree of conservation within than between lineages. However, at the current stand of our knowledge, we cannot tell if these sequences are maintained on mini-chromosomes or core-chromosomes in other isolates of the same lineage.

We also rephrased our conclusion to point out that mini-chromosomes either emerge independently in host-specific lineages **or individual isolates.**

We believe that further analysis (e.g. analysis of mini-chromosomes of multiple isolates of specialized lineages) is a major undertaking that is beyond the scope of the present manuscript.

*2. Why were only 772 non-redundant, mini-chromosome encoded proteins included in the Pfam domain search (page 31, line 375)?*

Added following sentences in material and methods section for clarification:

“We used a total of 772 non-redundant, mini-chromosome encoded proteins for the analysis. These contain all mini-chromosome encoded proteins after removal of duplicated, identical sequences.”

*3. With the emphasis on secreted proteins and their possibility to serve as effectors, 50, 8, and 2 secreted proteins were found on the mini-chromosomes in the three host specific lineages (page 32, lines 369-398). Are any of these actually expressed during infection? Similarly, there were 7 MAX-effectors on the mini-chromosomes (page 34, lines 407-409) – are any of these expressed?*

We do not have transcriptome data for the isolates used in this study.

However, we think that expression of specific proteins during infection is beyond the questions addressed in this paper. We demonstrate the reproducibility and scalability of mini-chromosome isolation sequencing and provide evidence that core-genomic, structural rearrangements are associated with mini-chromosome emergence. Adding protein or mRNA expression data does not add that much to the main message of this manuscript.

*4. It would be nice to include a description of what was in the 392 kb region separating the regions of synteny around the ACE1 locus within FR 13. Is this mostly repetitive sequence?*

We added several new analyses to the manuscript that we hope will satisfy the reviewer. Specifically, we analysed all regions that are associated with the major structural rearrangements, including the mini-chromosome of FR13 to the paper. We analyse gene and repeat content within those regions. This analysis strengthens our hypothesis that repeat-rich regions are associated with structural changes and mini-chromosome emergence. However, we did not find anything out of the ordinary in the 392 kb region. Gene- and repeat content are both average in this region.

*5. Additionally, what is encoded within the 761 kb and 164 kb mini synteny regions around the ACE1 locus? Is there anything specifically that could be contributing to host-specificity?*

We identified one copy of the well characterized effector AVR-Pik, the Ace1 secondary metabolite cluster, and one potential MAX-effector in the syntenic region of FR13. For AVR-Pik and Ace1 their contribution to virulence or avirulence are well documented and clearly indicate a role in host specialization. For the predicted MAX-effector this is possible as well, but still unclear at the moment. We did not identify other genes on the mini-chromosome for which a role in host adaptation is published.
