# Supplementary material for: Genomic rearrangements generate hypervariable mini-chromosomes in host-specific isolates of the blast fungus
Source: PLoS Genet. 2021 Feb 16;17(2):e1009386. doi: 10.1371/journal.pgen.1009386 (PMC7909708; doi:10.1371/journal.pgen.1009386)
Supplement: Attachment — Submitted filename: Response to reviewers_r2.docx [file pgen.1009386.s026.docx]

**Response to reviewer comments for the manuscript “Genomic rearrangements generate hypervariable mini-chromosomes in host-specific isolates of the blast fungus” by Langner et al., 2020.**

We thank the editor and reviewers for their comments. We note that the reviewers acknowledged that we addressed many comments and that the article is complementary to Peng et al. as expected from the PLOS “Scooping” policy. We also note that Peng et al. was an exclusively genome analyses paper and did not include follow-up wet lab experiments.

In reponse to the reviewer comments, we have continued to improve and expand our paper and have made the following revisions.

**Reviewer #1:**

The authors have well addressed most of my questions/comments.

We are pleased that the Reviewer considered that we addressed most of their questions/comments.

I still do not fully agree the following statement and appreciate the consideration about some levels of similarity between mini-chromosomes.

Here is the statement in the revision: “The difference in sequence similarity between core- and mini-chromosomes supports the hypothesis that mini-chromosomes represent isolate-specific genomic entities, possibly involved in host specialization and suggests that mini-chromosomes emerged independently in host-adapted lineages or individual isolates.”

We took stock of the comment and toned down our initial conclusion about lineage-specificity to better reflect the data presented in this manuscript, especially following the revision. We now conclude that mini-chromosomes might emerge in individual isolates rather than lineages. We changed this statement in the title and throughout the manuscript. We further rewrote the text to address the Reviewer’s concern:

“Comparative analyses of the mini-chromosomes sequences revealed that they are highly variable between isolates indicating that they may have emerged independently or that they have diverged rapidly after an ancient emergence event. Comparative analyses with core chromosomes indicate that the mini-chromosomes partially originate from rearrangements and segmental duplications of core-chromosomes.”

We further added analyses addressing lineage specificity of mini-chromosome sequence and genes encoded on mini-chromsomes. Specifically, we analysed conservation of mini-chromosomal sequences in 107 genome assemblies from 10 genetic lineages (incuding isolates from 13 host plants and one *M. pennisetigena* and six *M. grisea* isolates). The results of this analysis suggests that some regions of the FR13 mini-chromosome are conserved across lineages by partial reintegration into core-chromosomes. On the other hand, the US71 and CD156 mini-chromosome seem to be largely isolate specific.

Additionally, we discuss mini-diversity (in size) within lineages as observed since the 1990s and variability of the FR13 mini-chromosome between rice-infecting isolates. Based on this intra-lineage diversity we deem it more likely that mini-chromosomes emerge independently.

**Reviewer #2:**

The authors have address all my concerns. No

We are pleased that the Reviewer considered that we addressed all of their questions/comments.

**Reviewer #3:**

In this revised submission concerning mini-chromosomes in Magnaporthe oryzae, the authors indicate that their original submission was in respect to the PLOS “scooping” policy and have addressed some of the original concerns in this resubmitted manuscript. The manuscript has improved with additional analyses, and does provide complementary information in support of the Peng at al publication in regards to the “scooping” policy.

We are pleased that the Reviewer considered that the manuscript has improved with additional analyses and that it is complementary to Peng et al.

As mentioned in the previous review, the manuscript adds little new information regarding the general biology of fungal supernumerary chromosomes. In the absence of additional experiments, it is difficult to assess the contribution of these chromosomes to virulence, in particular host specificity.

Questions from previous review:

- Can mini-chromosomes be lost?
- Do they influence virulence / host-specificity?
- Are mini-chromosome encoded effectors expressed during infection?

*Are any of these mini-chromosomes able to be lost? Do they influence the host-specificity of each of the lineages?*

We acknowledge that there are still open questions regarding the biology of mini-chromosomes in *Magnaporthe oryzae*. However, we want to point out again that the novelty of our study should be evaluated based on the PLOS “Scooping” policy and with regard to the complementary study by Peng et al. which provides similar conclusions as our study.

Nevertheless, we attempted to address some of the reviewer concerns by investigating effector expression and therefore a virulence function of mini-chromosomes by RNA-seq and qRT-PCR. Unfortunately, the relevant isolate, FR13, was not virulent under our experimental conditions and it was not possible to generate robust expression data. We suspect that long term cultivation of this isolate under laboratory conditions led to decreased virulence. Please note that in this study we focused on analysing the isolates previously sequenced by Chiapello et al. 2015 (GEMO database http://genome.jouy.inra.fr/gemo) to clarify that three of four of the isolates they sequenced carry (overlooked) mini-chromosomes. We felt that would be a good service for the community. Thus the choice of the lab strain FR13 was not driven by pathology but rather our aim to build on community resources.

To address the reviewers concerns, we added analyses regarding the conservation of mini-chromosomal sequences and mini-chromosome encoded genes across lineages. The results of this analysis shows that the FR13 mini-chromosomes is partially reintegrated into core-chromosomes in various lineages. The host-specificity determining effector genes for AVR-PikD and AVR-PikA are excluded from these reintegrations in non-rice infecting isolates. This observation strengthens our conclusion that genomic rearrangements that involve mini-chromosomes influence host-specificity.

To further address the reviewers concerns we added extensive discussion regarding the contribution of mini-chromosomes to virulence. It is well documented in the literature that *M. oryzae* mini-chromosomes influence virulence and host-specificity (see new Discussion paragraph). We also discuss rearrangements between mini- and core-chromosomes and the associated loss of mini-chromosomes in greater detail.
